# Urdu translation of Shoulder Pain and Disability Index (SPADI) and its validity and reliability on adhesive capsulitis patients

**DOI:** 10.1186/s12891-022-05182-3

**Published:** 2022-03-08

**Authors:** Ammara Munir, Mehwish Ikram, Syed Shakil Ur Rehman

**Affiliations:** Faculty of Rehabilitation and Allied Health Sciences, Riphah International University Islamabad, Lahore Campus, Lahore, Pakistan

**Keywords:** SPADI, Shoulder pain, Adhesive capsulitis, Validity, Reliability, ICC_2,1_

## Abstract

**Background:**

Excessive scar tissues around the shoulder are the results of shoulder pathologies that lead to pain and disability. The Shoulder pain and disability index (SPADI) is used to measure the level of pain and disability in patients with shoulder pathology. SPADI is translated into Urdu and its validity and reliability are measured on patients with adhesive capsulitis.

**Objective:**

The study was aimed to translate the SPADI in Urdu and to evaluate its reliability and validity in patients with shoulder adhesive capsulitis.

**Methods:**

Translation of SPADI in Urdu was conducted by applying the standardized process. Two forward translations in Urdu were made T1 and T2 by bilingual translators. Urdu version of SPADI was drafted after experts’ opinion. Two Backward English translations of Urdu SPADI were made BT1 and BT2 and the back translation was finalized by the consensus of all experts. After this process of reviewing by the professional experts, 3rd version of Urdu SPADI was drafted. The Final version was drafted after its application on 10 patients. Its reliability and validity were tested on 150 patients with shoulder adhesive capsulitis.

**Results:**

Content Validity Index was good with values of each item > 0.85. For Test–retest reliability, the Intraclass correlation coefficient (ICC_2,1_) was measured with a value of 0.89 which showed good Test–retest reliability. The internal consistency and reliability of SPADI were calculated by Cronbach’s alpha for a total score with a value of 0.94. Construct validity and Concurrent validity were determined. In Construct validity, factor analysis of Urdu SPADI showed two factors and a cumulative variance of 75.443%.

**Conclusion:**

It was concluded that the Urdu version of SPADI is a valuable translation that is a valid assessment tool for patients with shoulder adhesive capsulitis. It has good validity and test–retest reliability.

## Introduction

Adhesive capsulitis is a painful condition in which the range of motion; active and passive is progressively decreased at the glenohumeral joint. Frozen shoulder term is also used for this condition; it was coined by Codman for no specific pathology [1, 2]. It has many other names e.g. Peri-arthritis of shoulder and scapula, Duplay disease, tendinitis of short rotators. Gradual onset of pain of several months referred to as the deltoid, night pain is common and difficulty in sleeping on the same side is observed [2]. Adhesive capsulitis affects females mostly between the ages of 40–60 years. Multiple conditions increase the risk for the development of Adhesive capsulitis; these conditions may be systemic or neurologic. It is 2–4 times more common in diabetics [3]. Clinically there are three stages of this disorder, the first is painful, the second is frozen, and the third is the recovery stage. The first stage may remain for 2–9 months, the second stage from 4–12 months in which pain decreases but movement restricts, and in the recovery stage, the range of motion starts to improve after 12 to 42 months [4].

The Shoulder pain and disability index (SPADI) was developed to measure the pain and disability of the shoulder. The original version was made by Roach and her colleagues in 1991[5]. It contains 13 items, 5 for pain and 8 for disability. In the original version of SPADI, the pain was measured from the visual analogue scale which was modified later by William in 1995[6]. The modified version was easy to use as compared to the original [5–7]. A significant correlation of SPADI with NPRS and pain changes immediately after treatment as compared to disability. It showed good responsiveness [8].

SPADI was translated in different languages for the feasibility of assessors and the population of the respective language. It has been translated into different languages e.g. Brazilian, Arabic, Dutch, German, Thai, Italian, Chinese, Turkish, Hindi, and Spanish [9–18]. As this scale has standard validity and reliability, therefore time demands to cross-culturally adapt it into a regional Pakistani language (Urdu) so this tool was selected for linguistic study. The original SPADI was chosen for translation and linguistic validation was done to facilitate the use of SPADI in regional language for Urdu-speaking patients with shoulder adhesive capsulitis.

Some patients in Pakistan find it difficult to understand an English questionnaire due to the language barrier and moderate rate of literacy. Pakistan has one national language (Urdu) and there are five provinces, every province has its local language. This restricts the implementation of SPADI as a clinical assessment tool in Pakistan. Thus, linguistic validation study in regional Urdu language is compulsory to facilitate its application for the local population. In a short communication by Haroon et al. (2019), an Urdu version was reported without the inclusion of authors of SPADI. Only test–retest reliability was checked, there was no content, construct and convergent validity [19]. Thus, the work was not included in the systematic review carried out by Furtado et al. [20]. As per the authors knowledge there is no Urdu version of SPADI with Standard International Guidelines. Therefore, a standardized study was aimed to cross-cultural adapt and evaluate the reliability and validity of the SPADI that is translated into the national language of Pakistan (Urdu).

## Material and methods

### SPADI scale

The Shoulder Pain and Disability Index (SPADI) is a self-administered tool for shoulder joint assessment having a total of 13 items classified as pain and disability subscales. By the combination visual analogue scales rating and the means of pain and disability subscales, a total score for both ranging from 0 to 10 is formulated. The SPADI was purposed to find out the influence of shoulder conditions in terms of pain and disability scores including recent measures and any change observed with specific time duration [5, 6].

### Translation process

Translation and cross-cultural adaptation methods were according to the American Association of Orthopedic Surgeons (AAOS) Outcomes Committee guidelines [21]. Measurement properties were according to COSMIN (Consensus-based Standards for the selection of health Measurement Instruments) Guidelines [22].

### Content validity

Several content validity indexes (CVI) have been developed to measure the content validity of instruments. We have used 4 points ordinal content validity index developed by Waltz to measure the content validity of U-SPADI. The content validity index consists of four categories that are relevance, clarity, simplicity, and ambiguity. Content validity according to Waltz and Bausell’s method was also determined by six physical therapists; each of the categories of the CVI was evaluated on four points Likert scale [23–25].

### Participants and data collection

Adhesive capsulitis patients were taken from the Physiotherapy Department at the Qari Hospital Okara, Pakistan. The study was started after the approval of the ethical committee from Riphah International University with the reference number REC/RCRS/19/1019. Permission was taken from the author of SPADI.

Inclusion criteria of patients were ranged from age 20–60 suffering from adhesive capsulitis with decreased active and passive range of motion. Patients must understand the Urdu language. Patients with a history of shoulder surgeries, neck pathologies with symptoms radiating to the upper extremity, and neuropathy were excluded. The institution issued the ethical approval and a consent form was obtained from all the individuals who participated in the study.

For Concurrent/ Convergent validity, U-SPADI was correlated with the Numeric Pain Rating Scale (NPRS) [26], Visual Analogue Scale (VAS) [27], and Quick Disabilities of Arm, Shoulder, and Hand (Quick DASH) [28] and Short Form Survey (SF-12) [29].

### Statistical analysis

Data analysis was done by using the SPSS version 25 (IBM, USA). Internal consistency was determined by using Cronbach’s alpha. The scores that are ranged from 0.50 to 0.69 are considered as poor, 0.70 and 0.79 acceptable, 0.80 and 0.89 good, and the value of Cronbach’s alpha that is > 0.90 is considered excellent [30].

Test–Retest reliability was measured with Intraclass correlation (ICC) on week 1 and week 2. ICC values are estimated at 95% confidence interval, values which are less than 0.5 indicate poor, 0.5–0.75 indicate moderate, between 0.75 indicate good and 0.90 excellent reliability [31].

Convergent and concurrent validity was determined by the correlation coefficients that vary from -1 to + 1. Negligible correlation ranged from 0.00–0.10, weak correlation ranged from 0.10–0.39, moderate correlation range is 0.40–0.69, strong correlation range is between 0.70–0.89 and very strong correlation is taken between 0.90–1.00 [32].

Factor analysis was done to find out the construct validity of the tool. Kaiser–Meyer–Olkin (KMO) measure of sampling adequacy and Bartlett’s test of sphericity was used to indicate the proportion of variance in the variables that might be caused by underlying factors. KMO ranged from 0 to 1, while 0.50 is accepted as reasonable for factor analysis [33].

## Results

### Translation process

**Fig. 1:**
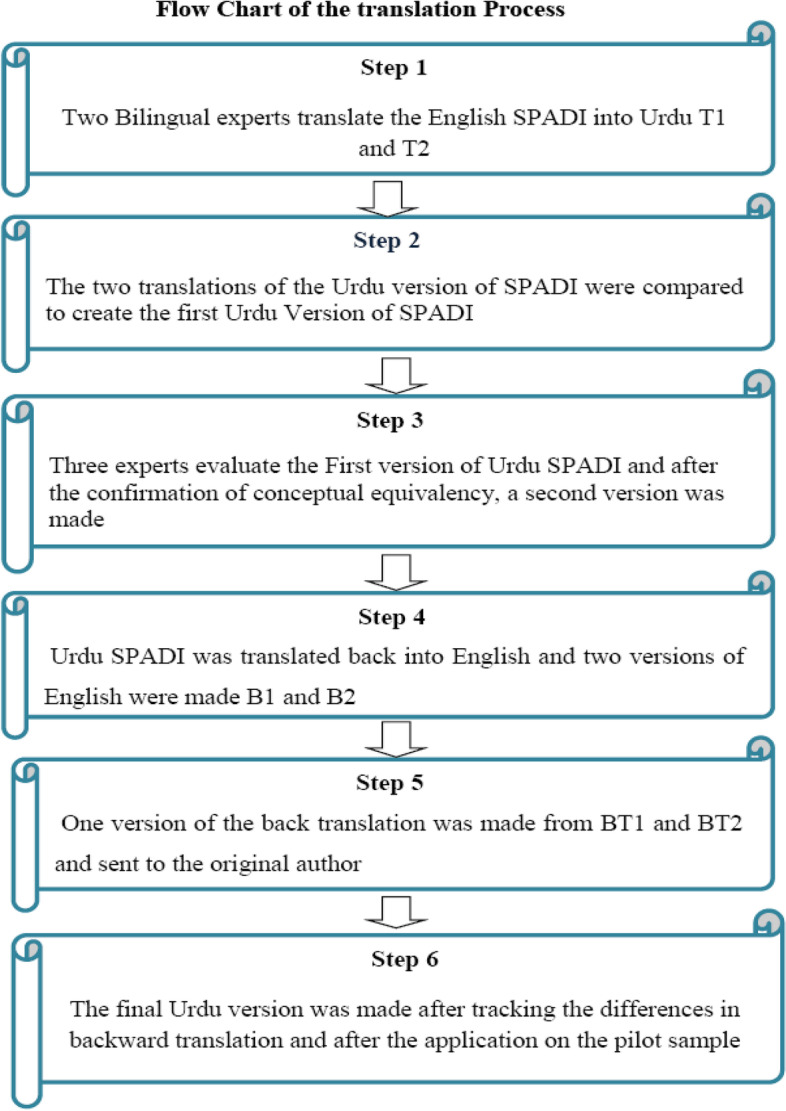
Flow chart of the translation process

Translation permission was taken from the author of SPADI. Figure 1 describes the translation process**.** Forward Translations of SPADI were done in Urdu by two bilingual translators (native speakers) and version 1 was drafted by two translated versions T1 (by translator1who have no medical background) & T2 (by translator 2 who know the scale). Version 1 was drafted by a neutral person. Version 1 was reviewed by the expert committee (three expert reviewers who have experience of more than 10 years in the physiotherapy profession) and Version 2 was drafted after modifying the required words to maintain the original concept of the questions.

Version 2 of Urdu SPADI was translated back into English; two translations were done according to international guidelines [21]. After experts’ review, backward translation was finalized and sent to the Author of SPADI.

3rd version was drafted after tracking the differences in the backward and original versions and a pilot study was done on 10 patients. The final version of Urdu SPADI was drafted.

### Cross-cultural adaptation of Urdu-SPADI

No major issues were experienced during the translation process into the Urdu language. Although, some phrases were discussed such as “at its worst,” “touching the back of your neck,” and “back pocket.” The questionnaire was translated without noticeable difficulties excluding a few words having synonyms, such as the starting questions Urdu word “bay inteha” may be assumed as excessive or intense. Similarly, the initial question in the pain subscale is the Urdu word “shadeed tareen” can be considered as unbearable or worse as the backward translator translated it. After discussion, it was decided that the word “bay inteha” will be suitable to translate “at its worst.” To simplify the complex context, a few words have been replaced during translation as ‘‘the back’’ was translated in Urdu word as “uqbi” by the first translator which was changed to another Urdu word “peechay” in relevant questions except for “washing your back” where the back was translated by an Urdu word “kamar”. The Urdu language is derived from Hindi, Persian, and Arabic and a big influence by English is observed on the regionally spoken Urdu language. All these factors were considered while designing the final version of SPADI. There was difficulty in the translation of the following items as touching the back of your neck and lifting a heavyweight of 10 pounds.

Percent agreement was calculated on the 3^rd^ version of Urdu-SPADI. There was perfect agreement shown between the experts on the translation process. (Table 1).

**Table 1:** Percent agreement of the Experts on 3^rd^ Urdu-SPADI version

Sr.No	Urdu-SPADI	Expert 1	Expert 2	Expert 3	Percent Agreement
**1.**	**PAIN**				
	Question 1	3	3	3	100%
	Question 2	3	3	3	100%
	Question 3	3	3	3	100%
	Question 4	3	3	3	100%
**2**	**DISABILITY**				
	Question 5	3	3	3	100%
	Question 6	3	3	3	100%
	Question 7	3	3	3	100%
	Question 8	3	3	3	100%
	Question 9	3	3	3	100%
	Question 10	3	3	3	100%
	Question 11	3	3	3	100%
	Question 12	3	3	3	100%
	Question 13	3	3	3	100%

During the process of translation (3^rd^ version), 3 experts opinion on the choice of words were evaluated on 3-point Likert Scale. The points chosen were, 3: Complete agreement, 2: Partial agreement and 1: No agreement

### Pilot study

The pilot study was done on the 10 healthy participants who have no shoulder pathology and on 10 adhesive capsulitis patients. Table 2 describes the demographic data of the pilot study. Discriminant validity was determined by an independent t-test, comparing the means of healthy and patients’ scores and it showed a significant difference. It showed that Urdu-SPADI was made for the assessment of shoulder pathologies. 10 patients, who were able to understand both English and Urdu, filled out English and Urdu SPADI questionnaires with a difference of one day. And the results were almost the same.

**Table 2:** Descriptive statistics of 10 individuals with adhesive capsulitis were included in the pilot testing

S.No	Age (years)	Gender	Side of Shoulder	BMI	Educational Status	U-SPADI
1	35	Female	Right	24.2	Graduated	61.5%
2	39	Female	Left	26.5	Metric	82.3%
3	45	Male	Left	30.4	Metric	70%
4	34	Female	Left	25.1	Intermediate	73.8%
5	38	Female	Left	24.5	Intermediate	60.7%
6	39	Female	Left	32.1	Graduated	64.6%
7	44	Male	Right	23.3	Graduated	81.5%
8	42	Male	Left	25.4	Graduate	80.7%
9	33	Female	Right	27.3	Graduate	83%
10	37	Male	Right	21.2	Intermediate	70%

*Abbreviations*: *BMI* Body Mass Index, *U-SPADI* Urdu Shoulder Pain and Disability Index

### Content validity

Content validity of the final version was determined according to Waltz and Bausell’s method by six physical therapists (who were not involved in process of translation and have clinical experience of > 5 years), it ranging from 0.87 to 0.95 [23–25].

### Reliability and validity

The demographic characteristics of the 150 patients with adhesive capsulitis are listed in Table 3. Convergent/Concurrent validity was determined by correlating the NPRS, VAS, Quick DASH and SF-12. NPRS, VAS, and Quick DASH showed moderate correlation while SF-12 weak correlation. Values are listed in Table 4.

**Table 3:** Descriptive statistics, Demographics of 150 patients

Descriptive Statistics of 150 Patients	
Male (n, %)	67 (44.7%)
Female (n, %)	83 (55.3%)
Age, (Mean ± S.D)	
Male	48.23 ± 5.19
Female	50.36 ± 7.58
^a^BMI (Mean ± S.D)	
Male	26.2 ± 3.5
Female	25.5 ± 4.6
The affected side of the shoulder	
Right	54 (36%)
Left	91 (60.7%)
Both	5 (3.3%)
Educational status	
Below Metric	48 (32%)
Metric/Intermediate	87 (58%)
Graduate	15 (10%)
U-SPADI (Week 1) Mean ± S.D	
Pain	34.5 ± 3.97
Disability	48.9 ± 6.60
U-SPADI (Week 2) Mean ± S.D	
Pain	31.88 ± 4.01
Disability	42.12 ± 5.53

^a^BMI: Body Mass Index

**Table 4:** Internal consistency, Test–retest reliability and validity on 150 Adhesive Capsulitis patients

Internal Consistancy: Cronbach alpha	
Pain (Subscale)	0.882
Disability (Subscale)	0.915
Urdu-SPADI (Total)	0.940
Test–Retest Reliability: Intra Class correlation (ICC)	
Pain (Subscale)	0.95
Disability (Subscale)	0.95
Urdu- SPADI (Total)	0.89
Pearson Correlation of week 1 and week 2 readings	
Pain (Subscale)	0.914
Disability (Subscale)	0.924
Urdu-SPADI	0.957
Convergent /Concurrent Validity (Correlation)	
VAS	r = 0.68
NPRS	r = 0.65
Quick Dash	r = 0.79
SF-12	r = 0.40

*Abbreviations*: *VAS* Visual Analogue Scale, *NPRS* Numeric Pain Rating Scale, *Quick Dash* Quick Disabilities of Arm, Shoulder and Hand, *SF-12* 12-Item Short Form Survey

For factor analysis accurate sample size was required which follows KMO criteria of sampling adequacy. After factor analysis for a sample size of 150 patients, KMO was as follows 0.864 and *p*-value is taken less than 0.05 which shows the test is highly significant [33].

**Fig. 2:**
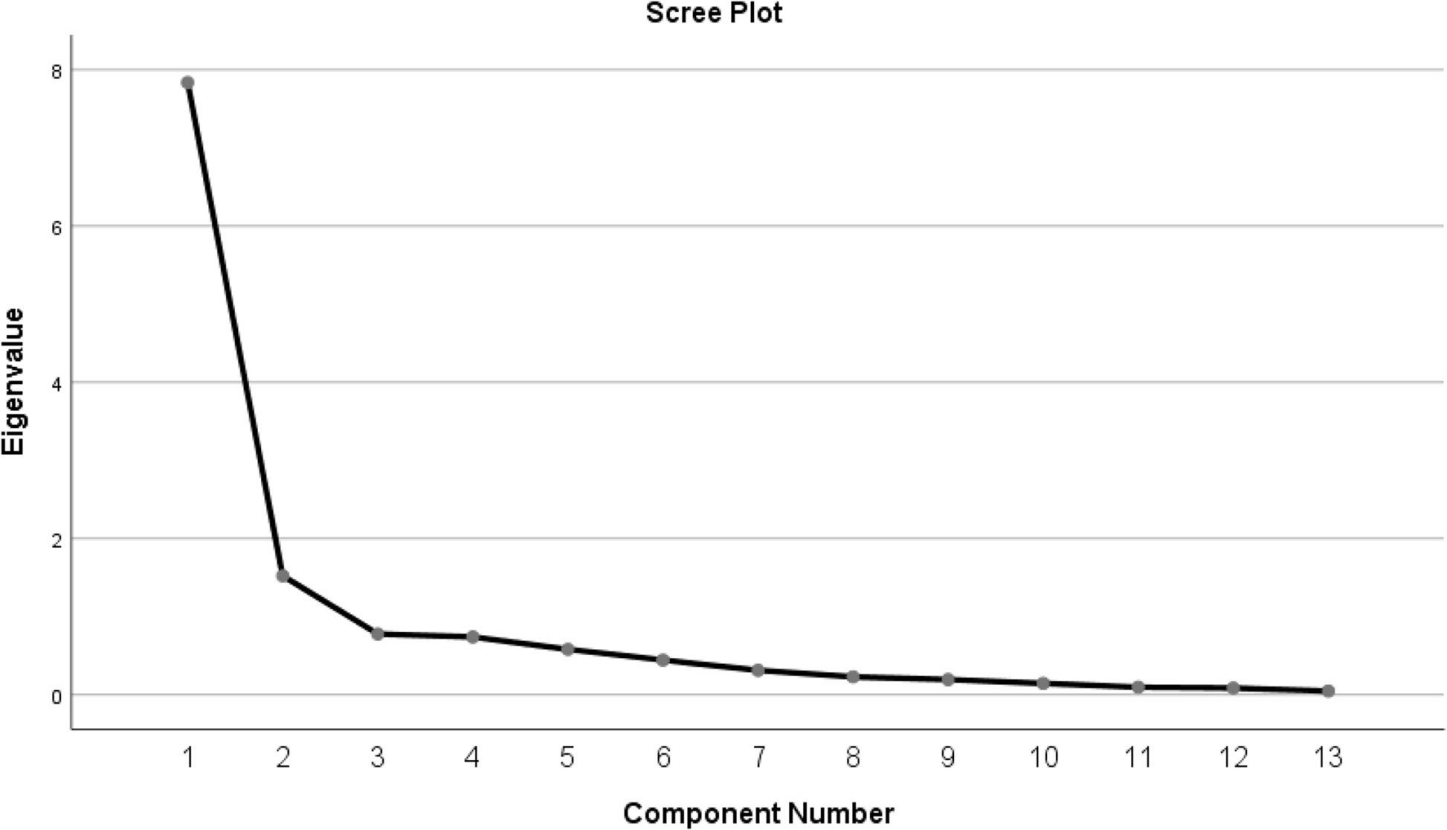
Scree plot showing the number of components

**Fig. 3:**
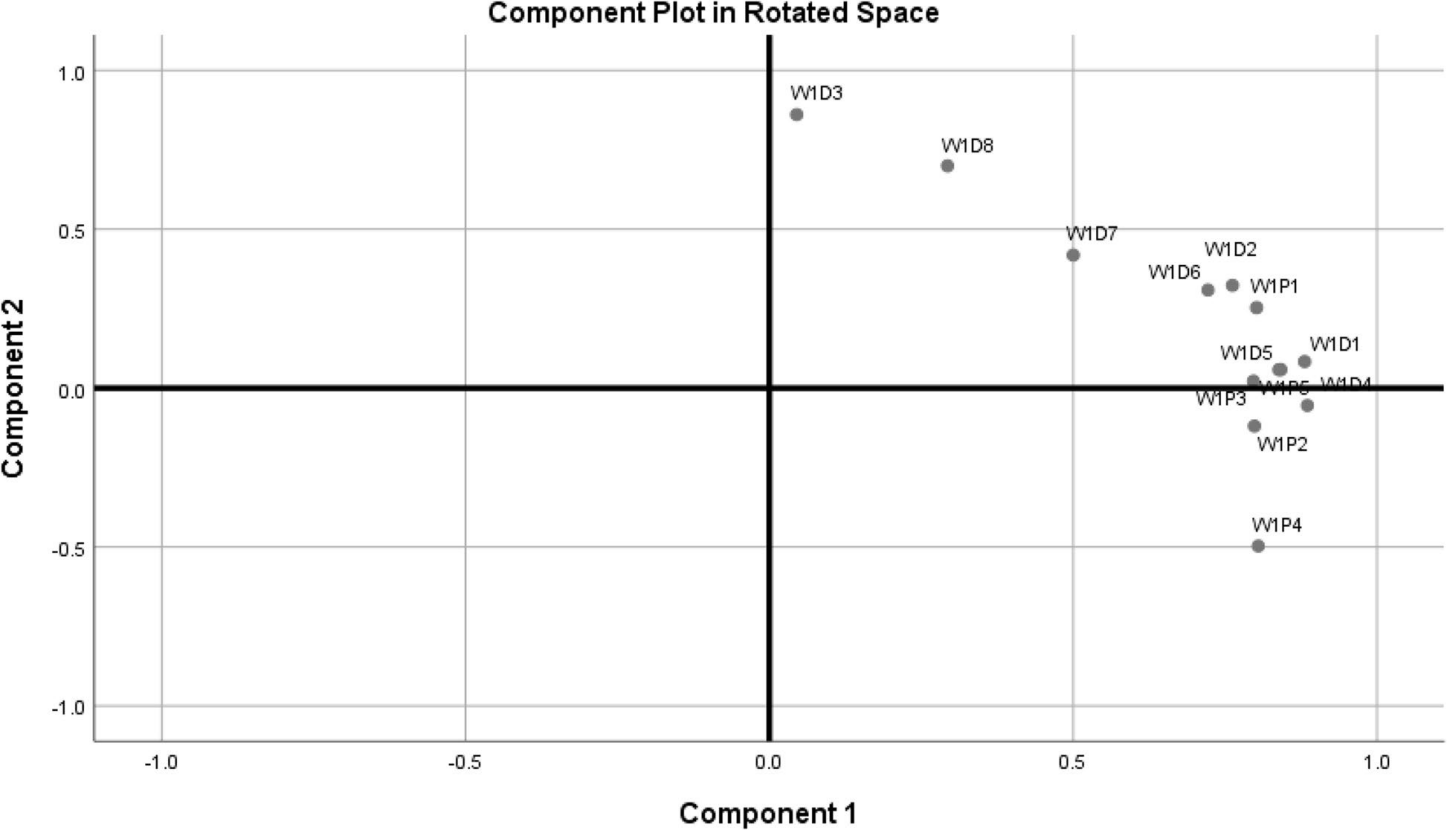
The component plot in rotated space. *Abbreviations: W1: Week 1; P: Pain; D: Disability; Numbers: 1, 2,3,4,5,6,7,8, indicate the question numbers of the SPADI

The results of factor analysis showed that the total variance of Urdu SPADI is described by two components, component no.1 shows 59.88% variance, component no.2 indicates 15.56% variance and cumulative variance of both components is 75.44%. A scree plot was found to have 2 components having an eigen value greater than 1 out of 13 items (Fig. 2). Hence, the two components will be the ones on which all items will be loaded. The factor analysis showed 75.44% total variance of all items (Fig. 3).

## Discussion

A standardized step-wise translation process was followed. Cronbach alpha (α) of pain is 0.88, for disability it was 0.91 and for total Urdu SPADI, it was 0.94. Week 1 and week 2 correlations were calculated and ranged from 0.84 to 0.95. In this study, the Intraclass correlation of each question of week 1 and week 2 was determined and it was ranged from 0.90 to 0.97. Cronbach alpha values are used to measure the internal consistency of the data. Urdu SPADI was correlated to determine the Convergent/Concurrent validity with Numeric pain rating scale (NPRS) [26], Visual Analogue Scale (VAS) [27], Disabilities of Arm, Shoulder, and Hand (Quick DASH) [28]. This showed a positive moderate correlation with all scales except SF-12 [29] where a weak positive correlation was found.

The Brazilian version of SPADI showed internal consistency values 0.70 to 0.90 and item ICC values were ranged from 0.64 to 0.92 [9]. Turkish version of SPADI was made in 2008 and its validity was determined by using different scales SF-36, DASH and found a significant relationship with them. Internal consistency was good, 0.83 for pain and 0.83 for disability. Turkish version was a reliable and valid tool for the assessment of shoulder pathology [16]. The German version of SPADI also showed a stronger relationship with SF-36, DASH, and ASES. The reliability of German SPADI was higher than original English SPADI and ICC values were also higher as compared with English [12]. Spanish version of SPADI applied on different disorders of the shoulder showed a strong positive correlation between the Spanish SPADI and DASH and moderate correlation with the VAS and weak positive correlation with the SF-12. ICC_2,1_ Values were higher 0.91 for total scores [18].

The Thai version of SPADI was used for validity and reliability in 2015 [13]. A total of 44 patients participated in that study, including mostly females (68.2%). Thai SPADI was correlated with DASH and SF-36. Results identified the value of Cronbach’s alpha coefficient of the Thai SPADI for pain was 0.92, for disability was 0.94 and total scale was 0.95. Thai SPADI has good reliability and validity for the measurement of shoulder disability in the Thai population [13].

Psychometric testing of the SPADI in the Tamil-speaking Indian population was done in 2012 [34]. The results concluded the reliability of the translated Tamil SPADI and its subscales by calculating the Intraclass correlation coefficient greater than 0.90. The internal consistency of the Tamil SPADI was calculated by Cronbach's alpha was greater than 0.95. The translated Tamil SPADI is convenient to implement and it has good reliability and validity to measure shoulder pain and disability in the south Indian population [34]. In 2019, the Nepali (SPADI-NP) version was drafted and its validity and reliability were determined. Participants filled SPADI-NP in two visits for assessments. Results showed that the internal consistencies were good for the pain subscale Cronbach’s *α* = 0.82 and for the disability subscale, the value of Cronbach’s *α* = 0.88. The test–retest reliability was also good for pain = 0.89 and disability = 0.96. The Nepali version of the SPADI showed excellent psychometric properties. It is designed for the measurement of shoulder pain and disability among patients presented with shoulder pain in Nepal for the research field as well as clinical practice [35]. The translated version of the Chinese SPADI showed high internal consistency (Cronbach’s *α* = 0.91). Test–retest reliability was high as calculated by the Intraclass correlation coefficient with the value of 0.87. The Chinese SPADI is a valid and practical tool that is designed to assess the level of pain and disability among the Chinese population having complaints of shoulder disorders [15].

In this study, only adhesive capsulitis patients have participated. We can also use this Urdu version of SPADI for patients with other shoulder complaints. Urdu SPADI can be used on large samples from different hospitals and cities.

## Conclusion

Translation of Shoulder Pain and Disability Index (SPADI) into the Urdu language was done. It was concluded that the Urdu translated version of SPADI has good reliability and validity in patients with shoulder adhesive capsulitis.

### Limitations

In this study, U-SPADI was only used for the assessment of adhesive capsulitis patients.

### Implications to physiotherapy

The Urdu version of SPADI will be beneficial for Urdu speaking population so that they may easily fill out a self-reported questionnaire.

It will be a convenient way to assess the patient complaints on its own. This scale is a valid tool for all the conditions of shoulder pathologies. So, Urdu-SPADI can be used for all problems that are related to the shoulder.

It will open new eras of research between clinicians and researchers worldwide.

## Data Availability

The dataset used and analyzed during the current study is available from the corresponding author on reasonable request.

## Abbreviations

SPADI: Shoulder Pain and Disability Index
AAOS: American Association of Orthopedic Surgeons
COSMIN: Consensus-based Standards for the selection of health Measurement Instruments
CVI: Content Validity Index
ICC: Intraclass correlation
NPRS: Numeric Pain Rating Scale
VAS: Visual Analogue Scale
Quick DASH: Quick Disabilities of Hand, Shoulder, and Arm
SF-12: 12-Item Short Form Survey
KMO: Kaiser–Meyer–Olkin
ASES: American Shoulder and Elbow Surgeons Shoulder Score
